# Virulent MDR *Edwardsiella tarda* from stinging catfish (*Heteropneustes fossilis*)

**DOI:** 10.1371/journal.pone.0340061

**Published:** 2026-01-30

**Authors:** Mst. Tachhlima Aktar, Bushra Benta Rahman Prapti, Aminur Rahman, Mst. Ayesha Siddyqua, Muhammad Tofazzal Hossain, Mahbubul Pratik Siddique

**Affiliations:** Department of Microbiology and Hygiene, Bangladesh Agricultural University, Mymensingh, Bangladesh; Nitte University, INDIA

## Abstract

Edwardsiellosis, caused by *Edwardsiella tarda*, is a highly pathogenic disease affecting both freshwater and marine fish, leading to mass mortality. This study encompassed the molecular detection, virulence and antibiogram profiling of *E. tarda* from *Heteropneustes fossilis* (stinging catfish). A total of 40 fish samples were collected from different fish farms in the Mymensingh district. Isolation of *E. tarda* was performed using selective media, followed by identification through morphological, cultural, and biochemical testing, and confirmed through polymerase chain reaction (PCR). Antibiogram was performed following the disc diffusion method, and virulence and antibiotic resistance genes were detected through PCR. 19 out of 40 fishes were positive for *Edwardsiella* infection and 32 *E. tarda* were isolated. PCR assays consistently amplified *gro*EL (623 bp), *gyr*B (415 bp), *etf*A (415 bp), and *etf*D (445 bp) genes, respectively. The prevalence of virulence genes was: *gad*B (17%), *muk*F (35%), *fim*A (10%), and *cit*C (12.5%). The antibiogram revealed that the highest resistance (90.63%) was found against cotrimoxazole and amoxicillin, whereas sensitive (100%) to gentamicin and meropenem. Among 32 isolates, 90.75% were found to be multi-drug resistant (MDR). Again, multiple antibiotic resistance (MAR) index analysis revealed the highest value of 0.61, and 93.75% of isolates have an MAR index above 0.2. Resistance gene distribution was observed as the highest (62.5%) isolates harbored the *tet*A gene and the lowest (21.88%) isolates harbored *qnr*B. It could be concluded that MDR and pathogenic potential *E. tarda* was present in stinging catfish, posing a potential threat to animals, humans, and the environment.

## 1. Introduction

Aquaculture has become one of the booming food production sectors worldwide, contributing over 223 million metric tons annually [1]. In Bangladesh, this sector plays a crucial role not only in food security [2] but also in employment and export earnings [3]. Among freshwater fishes, catfish are particularly suitable for semi-intensive systems. In Bangladesh, shark catfish (*Pangasius hypophthalmus*) and stinging catfish (*Heteropneustes fossilis*), locally named pangus and singh, are the most popular catfishes [4]. However, the sustainability of this sector is increasingly threatened by emerging infectious diseases, particularly those caused by antibiotic-resistant pathogens [5]. Factors, such as high-density farming, live aquatic species trade, climate change, and ecological shifts exacerbate the emergence and spread of diseases in aquaculture [3]. Failure to management these diseases contribute to rising economic losses and facilitate transmission between humans and fish [5]. Pathogens like *Aeromonas* spp., *Edwardsiella* sp., and *Vibrio* spp. considered major causes of high mortality and economic losses in freshwater aquaculture [6,7]. Among them, *Edwardsiella tarda* (*E. tarda*) has emerged as a significant concern due to its pathogenicity and antibiotic resistance profile [8].

*Edwardsiella tarda* is a Gram-negative bacterium, belong to the Enterobacteriaceae family and are commonly found in aquatic environments, Notably, it is recognized as an opportunistic human pathogen, capable of causing infection particularly in immunocompromised individual or following exposure to aquatic environment [9,10]. Historically, the genus *Edwardsiella* consisted of three species: *E. tarda*, a bacterium capable of infecting a wide range of hosts, including both fish and mammals; *E. ictaluri*, which causes enteric septicemia in catfish and outbreaks of edwardsiellosis in tilapia; and *E. hoshinae*, generally regarded as a commensal organism in birds and reptiles [11]. *Edwardsiella tarda* was first identified by Sakazaki and Murata in Japan in 1967 from human feces [12]. Edwardsiellosis, caused by *E. tarda,* is a gangrenous and emphysematous putrefactive disease in freshwater fish, with high prevalence in tilapia and catfish. Mortality in freshwater fish due to *E. tarda* ranges from 5% to 70%, with a prevalence of up to 70% [13]. Furthermore, mortality rates of 65% and 40% were observed in *Clarias gariepinus* and *Oreochromis niloticus* due to *E. tarda*, respectively [14]. Moreover, the economic burden is further aggravated by emergence of highly resistant strains [15].

Frequent exposer and interaction between aquatic and terrestrial bacterial strains facilitate the transfer of antimicrobial resistance determinants to pathogenic bacteria in fish [7]. Consequently, fish are recognized as potential reservoirs and disseminator of antimicrobial-resistant (AMR) bacteria and genes [16]. Antibiotics routinely used in aquaculture are prophylactic and therapeutic purposes. However, this practice accelerates the spreads of resistant genes via horizontal gene transfer mediated by genetic elements such as plasmids and transposons [17]. *E. tarda* has reported resistance to several commonly used antibiotics over time, raising serious public health concern [18,19].

Beyond its resistance capabilities, *E. tarda* possesses several virulence factors that enhance its ability to infect and persist within host organisms [20]. The virulence of *E. tarda* is considered a complex, multifactorial process that is not yet fully understood. Various potential pathogenic traits have been proposed to play a role in its infection process, such as the secretion of degradative enzymes, adhesins, the type III secretion system (T3SS), the type VI secretion system (T6SS), and the ability to survive and proliferate within phagocytes [21]. Many genes related to virulence in *E. tarda* have been identified and cloned, which include those encoding catalase (*kat*B), components of the T3SS (*ast*), T3SS regulator (*esr*B), extracellular apparatus of T6SS (evp), putative killing factor (*muk*F), fimbrial operon (*fim*A), glutamate decarboxylase (*gad*B), citrate lyase ligase (*cit*C), hemolysins (*hly*A), ankyrin-like proteins (*ank*), and the quorum sensing system (*lux*S) [22].

In Bangladesh, research on *E. tarda* is limited, with most studies focusing on basic identification and pathogenicity in different fish species. For instance, *Edwarsiella* has been reported in Catla (*Catla catla*), koi (*Anabas testudineus*), and tilapia (*Oreochromis mossambicus*) based on cultural, and biochemical characterization [23]. Additional investigations have examined pathogenicity through artificial challenge tests, assessed antibiogram susceptibility, and explored herbal sensitivity as potential treatment alternatives [24]. However, the molecular detection, virulence gene profiling, and genotypic correlation with antibiotic resistance of *E. tarda* remain underexplored in Bangladesh. This study aims to fill the gap by providing the first comprehensive molecular, phenotypic, and genotypic characterization of *E. tarda* strains isolated from aquaculture species in Bangladesh.

## 2. Method

### 2.1. Ethical approval

The whole protocol of this research work was ethically approved by the Animal Welfare and Experimentation Ethics Committee (AWEEC) of Bangladesh Agricultural University, Mymensingh-2202 (Approval No.: AWEEC/BAU/2024(5)/19b, date: 08/12/2024).

### 2.2. Sample collection and processing

Clinically suspected 40 apparently diseased stinging catfish (*Heteropneustes fossilis*) were collected based on characteristics external morphological signs from aquaculture farms of 3 different upazilas (Trishal, Muktagachha and Gouripur) of Mymensingh district, Bangladesh. Immediately after collection, all samples were kept in ice box to maintain cool chain and transported to the Bacteriology Laboratory, Department of Microbiology and Hygiene, Faculty of Veterinary Science, Bangladesh Agricultural University (BAU), Mymensingh. From each fish four tissues (skin, liver, kidney and intestine) were aseptically sampled, yielding 160 samples in total and examined for identification and isolation of *E. tarda* (S1 Table). Prior to dissection, fish were surface sterilized using sterilized distilled water to minimize contamination from skin flora. Tissues were excised aseptically using sterile forceps and scissors, homogenized in sterile phosphate buffer saline (PBS) with a stomacher blender.

### 2.3. Isolation and phenotypic characterization *E. tarda*

Primary enrichment of the samples was done using nutrient broth (HiMedia, India) and incubated at 37^°^C for 24 hours for primary enrichment. Cultures were streaked on Nutrient Agar (NA) plate (HiMedia, India) and incubated under same condition. Colonies that appear colorless, watery, smooth shaped colonies from NA were then streaked on MacConkey agar media (HiMedia, India) incubated at 37 ° C for 24–48 hours. Pale colour colonies grown on MacConkey agar media were then selected and further streaked on Salmonella-Shigella (SS) agar media (HiMedia, India) and Eosin methylene blue EMB agar (HiMedia, India) media to ensure purity. Small black centered colony from SS agar [25] and pale pink coloured, moist, glistening colonies on EMB agar were then streaked on the modified ET-Agar media and incubated at 28 °C for 24 hours [26].

The modified ET- agar was prepared with MacConkey agar base (40 g); yeast extract (1g); and additional agar (4.5 g) per liter. After autoclaving, two filter-sterilized solutions were added: (i) 100 ml of distilled water in which are dissolved glucose (2 g); sucrose (5 g); mannitol (5 g); xylose (5 g); L-lysine (10 g); sodium thiosulphate (6.8 g); ferric ammonium sulphate (0.8 g). (ii) 10 ml of colistin in distilled water (1 mg/ml). Colonies displaying clear to whitish with black center on ET-agar [27]. Pure colonies were selected for gram staining and microscopic observation and further identification*.*

### 2.4. Biochemical identification of the *Edwardsiella tarda*

Phenotypic identification was initiated with Gram staining and morphology was examined according to previously described method [28]. Purity was confirmed by consistent colony morphology on at least two different agar plates, and Gram staining showing a single cellular morphology. A confirmed pure colony was inoculated into sterile nutrient broth and incubated (18–24 h) to obtain fresh culture for biochemical tests and DNA extraction. All biochemical tests like catalase, sugar fermentation, Indole, Voges-Proskauer, Methyl-red, and motility tests were performed using fresh (18–24 h) pure cultures following the standard protocol [28]. The findings were compared with the previously published reports on the morphological and biochemical characterization of *E tarda*.

### 2.5. Determination of haemolytic activity on bovine blood agar

Hemolytic activity was tested as part of the identification process since *E. tarda* is reported to produce β-hemolysis on blood agar [29]. Isolated bacterial colonies were cultured on bovine blood agar media and incubated at 37 °C for 24 hours for observing hemolytic growth on this agar media.

### 2.6. DNA extraction and molecular detection of *E. tarda* by PCR assay

The presumed *E. tarda* isolates were subjected to DNA extraction and accomplished through boiling method [30]. Afterwards, the quantity and the quality assessment of the extracted DNA was done using NanodropTM (Thermo Fisher Scientific, USA). For molecular confirmation of *E. tarda,* PCR (polymerase chain reaction) was performed to detect *etf*A*, etf*D*, gyr*A (gyrase) with previously reported primer sequence. A newly designed primer sequence was developed targeting the *gro*EL gene. All the primers with their target gene and sequence and amplicon size are mentioned in Table 1.

**Table 1:** Phenotypic resistance, multiple antibiotic resistance (MAR) index, resistance genes, and virulence associated gene profile of *E. tarda* isolates.

Target gene	Primer Sequence (5’ to 3’)	Amplicon size (bp)		Thermal Profile				References
			Initial Denaturation	Denaturation	Annealing	Elongation	Final Elongation	
*etf*A	F: CGGTAAAGTTGAGTTTACGGGTG	415	94°C, 4 m	35 cycles			72°C, 7 m	[31]
	R: TGTAACCGTGTTGGCGTAAG			94°C, 30 s	57°C, 30 s	72°C, 30 s		
*etf*D	F: GGTAACCTGATTTGGCGTTC	445	94°C, 2 m	30 cycles			72°C, 7 m	
	R: GGATCACCTGGATCTTATCC			94°C, 20 s	72°C, 1 m	72°C, 1 m		
*gyr*B	F: GCATGGAGACCTTCAGCAAT	415	94°C, 4 m	30 cycles			72°C, 7 m	[32]
	R: GCGGAGATTTTGCTCTTCTT			94°C, 30 s	72°C, 1 m	72°C, 20 s		
*gro*EL	F: TGACTCTAAAGCCATTGC	623	94°C, 5 m	30 cycles			72°C, 5 m	This study
	R: TAACACGACCCTGAATGG			94°C, 30 s	72°C, 1 m	72°C, 30 s		
*muk*F	F: TTGCTGGCTATCGCTACCCT	357	94°C, 5 m	30 cycles			72°C, 10 m	[33]
	R: AACTCATCGCCGCCCTCTTC			94°C, 30 s	72°C, 1 m	72°C, 1 m		
*gad*B	F: TAAAGGGAAATAATGACGGCG	583	94°C, 5 m	30 cycles			72°C, 7 m	
	R: GGCTGTAGGTATCGGTTTTCG			94°C, 30 s	72°C, 1 m	72°C, 35 s		
*fim*A	F: CTGTGAGTGGTCAGGCAAGC	441	94°C, 5 m	30 cycles			72°C, 7 m	
	R: TAACCGTGTTGGCGTAAGAGC			94°C, 30 s	72°C, 1 m	72°C, 30 s		
*cit*C	F: TTTCCGTTTGTGAATCAGGTC’	596	94°C, 5 m	30 cycles			72°C, 10 m	
	R: AATGTTTCGGCATAGCGTTG’			94°C, 30 s	72°C, 1 m	72°C, 30 s		
*bla*_TEM_	F: ATCAGCAATAAACCAGC	516	95°C, 5 m	35 cycles			72°C, 7 m	[34]
	R: CCCCGAAGAACGTTTTC			95°C, 1 m	72°C, 1 m	72°C, 1 m		
*bla*_CTX-M_	F: ACGCTGTTGTTAGGAAGTG	759	94°C, 5 m	35 cycles			72°C, 10 m	[35]
	R: TTGAGGCTGGGTGAAGT			94◦C, 45 s	58◦C, 45 s	72◦C, 1 min		
*tet*A	F: GCTACATCCTGCTTGCCTTC	210	95°C, 5 m	35 cycles			72°C, 5 m	[36]
	R: CATAGATCGCCGTGAAGAGG			95°C, 30 s	55°C, 40s	72°C, 1 m		
*qnr*B	F: GGMATHGAAATTCGCCACTG	264	94°C, 4 m	35 cycles			72°C, 10 m	[37]
	R: TTTGCYGYYCGCCAGTCGAA			94°C, 1 m	55°C, 1 m	72°C, 1.30 m		
*aad*A1	F: TATCAGAGGTAGTTGGCGTCAT	484	94°C, 4 m	35 cycles			72°C, 7 m	[38]
	R: GTTCCATAGCGTTAAGGTTTCAT			94°C, 30 s	54°C, 40s	72°C, 45 s		
*aac*(6)’	F: TTGCGATGCTCTATGAGTGGCTA	482	94°C, 5 m	35 cycles			72°C, 7 m	[37]
	R: CTCGAATGCCTGGCGTGTTT			94°C, 45 s	55°C, 45 s	72°C, 45 s		

**Legends:** bp: base pair; m: minute; s: seconds; °C: degree Celsius

### 2.7. Antimicrobial susceptibility Testing

A set of 13 commercially available antibiotic disk used to determine the antibiotic susceptibility profile of isolated *E tarda* by disk diffusion method. The disk diffusion assay was performed following the Clinical and Laboratory Standard Institute [39] provided VET03 guidelines. Total 13 commercially available disks (Oxoid, Basingstoke, UK) *viz*., Amoxicillin (AMX, 10 µg), Streptomycin (S, 5 µg), Kanamycin (K, 30 µg), Ceftriaxone (CRO, 30 µg), Novobiocin (NV, 30 µg), Levofloxacin (LEV, 5 µg) Aztreonam (ATM, 30 µg), Gentamicin (CN, 10 µg), Nalidixic acid (NA, 30 µg), Meropenem (MEM, 10 µg), Azithromycin (AZM, 15 µg), Cotrimoxazole (COT, 25 µg) and Oxytetracycline (OT, 30 µg) were used. A fresh overnight culture of all isolates was to the 0.5 McFarland standards and swabbed evenly on Mueller-Hinton agar (HiMedia, India). Antibiotic disks were placed onto the agar plate gently. Plates were placed inversely and incubated overnight at 37°C. Diameter of the zone of complete inhibition (including disc diameter) was measured, which were then compared with the standard interpretative tables [39].

### 2.8. Molecular screening of virulence and antibiotic resistance genes

For virulence screening, four genes representing critical determinants of *E. tarda* pathogenicity in fish, were targeted: *muk*F *(*putative killing factor linked to stress survival), *gad*B (glutamate decarboxylase involved in acid resistance), *fim*A *(*fimbrial operon associated with bacterial adhesion), *cit*C *(*citrate lyaseligase contributing to metabolic adaptation and virulence regulation, including the type III secretion system). The antibiotic resistance genes (ARGs), viz., β-lactams (*bla*_CTXm_, *bla*_TEM_), tetracycline (*tet*A), quinolone (*qnr*B) and aminoglycosides (*aad*A1, *aac*(6)’), were also accomplished. The oligonucleotide sequence, target gene name, and expected amplicon size for each PCR assay are depicted in the Table 1.

### 2.9. PCR condition and amplification

All individual PCR reactions for the target genes were performed in a final reaction volume of 25 µL. The reaction mixture was composed Premix Taq (Takara Taq Version 2.0 plus dye, Takara Bio, Japan), respective forward and reverse primers, 100 ng DNA templates, and nuclease-free water up to 25 µL. Amplification profile of all genes are mentioned in (**Table 1**). The amplified PCR products were resolved by electrophoresis in 1.5% agarose gel (Sigma, USA) at 100 V for 30 minutes, stained with Safe Red DNA stain (Hebei SanshiBio-Tech Co. Ltd., China) and finally photographed using a Gel Doc 1000 documentation system (Bio-Rad Laboratories Inc., CA, USA).

### 2.10. Determination of Multi-drug resistance (MDR) patterns and Multiple Antibiotics Resistance (MAR) Index

Multi-drug resistance (MDR) patterns of all the isolates were assessed based on the recommendation of Magiorakos et al. [40], where, if any isolate show resistance against at least one agent of three or more antibiotic classes, then that would be considered as MDR. The Multiple Antibiotic Resistance (MAR) index, introduced by Krumperman [41], is a method used to assess and categorize bacterial isolates based on their resistance to various antibiotics. It helps determine the risk level of contamination, with high and low-risk groups based on the frequency of antibiotic resistance. According to Singh et al. [42], the MAR index is calculated by dividing the number of resistant antibiotics by the total number of antibiotics tested (intermediate-resistant isolates are considered sensitive). MAR index greater than 0.2 suggests that the bacterial isolates come from high-risk sources, while an index below 0.2 indicates low-risk contamination [43].

### 2.11. Data analysis

The SPSS statistic software version 25 was used to analyse data statistically. Chi-square test of independence was applied in comparing the prevalence/occurrence of virulence genes and ARGs with respect to sampling organs of fishes. A p-value ≤ .05 was considered as moderate to low significant and p-value ≤ .01 was considered as extremely significant. In order to evaluate the correlations among the virulence factors and resistance genes Spearman’s ranks correlation coefficient (r) was used to determine the p-value. A correlation was considered strong when |r| ≥ 0.8, moderate when 0.5 ≤ |r| < 0.8, weak when 0.3 ≤ |r| < 0.5, and negligible when |r| < 0.3, following the guidelines outlined by Spearman [44].

## 3. Results

### 3.1. Demographic data

Out of 40 suspected fish samples, 19 (47.5%) were detected positive for *E. tarda*. A total of 32 isolates were recovered, as multiple isolates were obtained from different tissue of same fish (intestine, gill, liver). Among the three sampling sites, Muktagaccha upazila exhibited highest prevalence rate (53%) followed by Trishal (50%) and Gouripur (45%). A summary of the distribution of positive fish and recovered isolates across locations and organs is presented in **Table 2**.

**Table 2:** Demographic data representing the percent occurrence of *E. tarda* in stinging catfish samples from various locations.

Location	Total fish sampled	Organ tested	No. of positive isolates	Isolate ID	Total positive fish (n)	Prevalence (%) of positive fishes
Trishal	12	Skin	4	TF2-S, TF5-S, TF7-S, TF11-S	6	50%
		Intestine	3	TF1-I, TF4-I, TF11-I		
		Liver	3	TF1-L, TF5-I, TF11-L		
		Kidney	0			
Muktagachha	17	Skin	5	MF3-S, MF4-S, MF6-S, MF8-S, MF16-S,	8	53%
		Intestine	6	MF3-I, MF6-I, MF8-I, MF10-I, MF12-I, MF16-I		
		Liver	4	MF4-L, MF6-L, MF8-L, MF12-L		
		Kidney	0			
Gouripur	11	Skin	3	GF2-S, GF9-S, GF10-S	5	45%
		Intestine	3	GF5-I, GF9-I, GF10-I		
		Liver	1	GF1-L		
		Kidney	0			
**Total**	**40**		**32**		**19**	**47.5**

### 3.2. Identification and prevalence of *E. tarda*

On modified ET agar*,* the isolates produced clear to whitish colonies with characteristic black centres. β haemolysis was observed as distinct transparent zone surrounding the colonies, indicating complete lysis of red blood cells. Additional cultural and biochemical characteristics are summarized in (S2 Table; S1 Fig).

**Fig 1:**
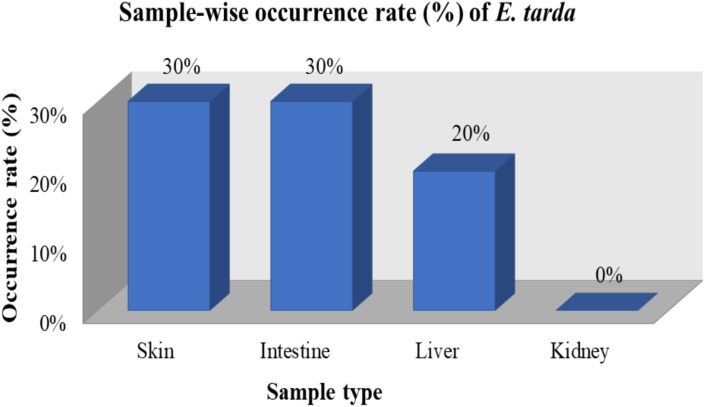
Sample-wise occurrence (%) of *E. tarda* in shing fish samples.

From 160 organ samples (skin, liver, kidney and intestine of 40 fish) 32 isolates (20%) were identified as *E. tarda* following cultural and biochemical characterization and later, confirmed by molecular detection. Location-wise, highest number of *E. tarda* was isolated from fishes of Muktagachha upazila (n = 15; 22.06%). Among the organ samples, the highest recovery was from skin and intestine (12/40 each), followed by liver (8/40), whereas no isolates were obtained from kidney samples (Table 3; Fig 1).

**Table 3:** Organ-wise distribution of *E. tarda* isolates recovered from different sampling areas of stinging catfish (*Heteropneustes fossilis*).

Total number of fishes	Organs	Total samples	Grand total of sample from individual area	No. of positive sample	Grand total of positive samples	Occurrence (%)	
						% with in organs	Total
Trishal (n = 12)	Skin	12	N = 48	4	10	33.33	20.83%
	Intestine	12		3		25	
	Liver	12		3		25	
	Kidney	12		0		0	
Muktagachha (n = 17)	Skin	17	N = 68	5	15	29.41	22.0%
	Intestine	17		6		35.29	
	Liver	17		4		23.52	
	Kidney	17		0		0	
Gouripur (n = 11)	Skin	11	N = 44	3	7	27.27	15.9%
	Intestine	11		3		27.27	
	Liver	11		1		9.09	
	Kidney	11		0		0	
**Total**			**160**		**32**		**20%**

### 3.3. Molecular detection of *E. tarda*

**Fig 2:**
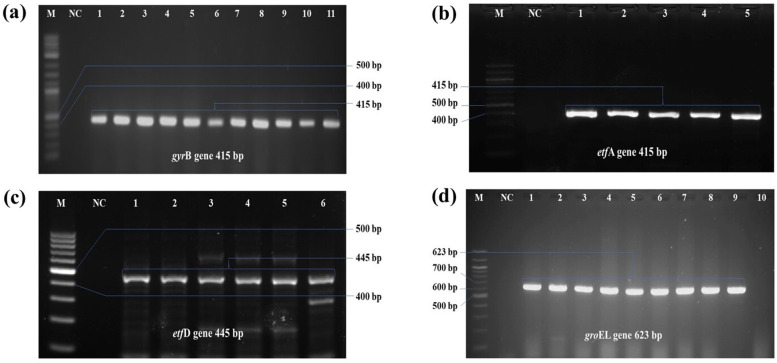
PCR amplification result of molecular detection of *E. tarda.* PCR amplification of species-specific primers used to detect *E. tarda* in stinging catfish samples. a) lane 1–11 positive amplicon at 415 bp for *gyr*B, b) lane 2–7 positive amplicon at 415 bp for *etf*A*,* c) lane 1–4, 6 and 9 positive amplicons at 445 bp for *etf*D*,* and d) lane 1–7 positive amplicon at 623 bp for *gro*EL gene. (M = 100 bp DNA ladder; NC-negative control)*.*

All 32 isolates were found positive for PCR, moreover, all isolates were positive for all the species-specific primer sets, used in the present study (Fig 2)

### 3.4. Antibiotic susceptibility patterns of the *E. tarda* isolates

**Fig 3:**
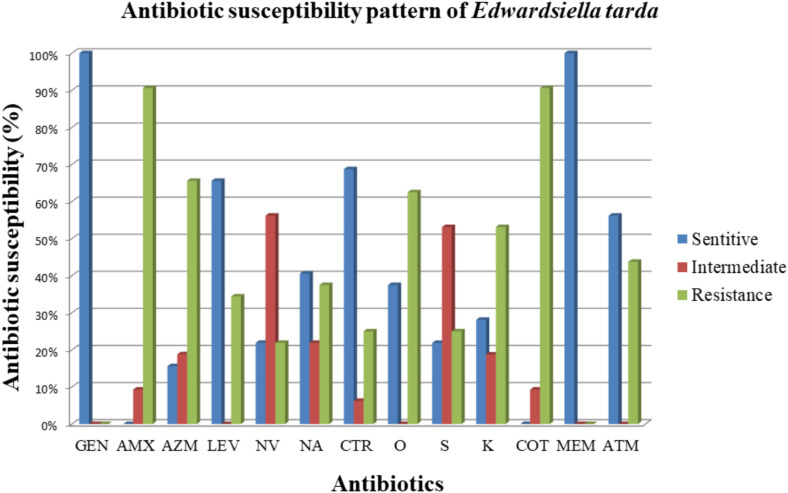
Antibiotic susceptibility pattern of *E. tarda* isolates. AMX: Amoxicillin; S: Streptomycin; K: Kanamycin; CRO: Ceftriaxone; NV: Novobiocin; LEV: Levofloxacin; ATM: Aztreonam; CN: Gentamicin; NA: Nalidixic acid; MEM: Meropenem; AZM: Azithromycin; COT: Cotrimoxazole; OT: Oxytetracycline.

Antibiogram revealed wide spread resistance among isolates. Most strains were resistance to Amoxicillin (93.75%) and Cotrimoxazole (90.63%), whereas Gentamicin and Meropenem were highly effective. Moderate resistance was observed against tetracycline and kanamycin, while intermediate resistance was common for streptomycin and novobiocin. Moreover, Ceftriaxone retained sensitivity against 75% of isolates (Fig 3).

### 3.5. Prevalence of antibiotic resistance determinants

**Fig 4:**
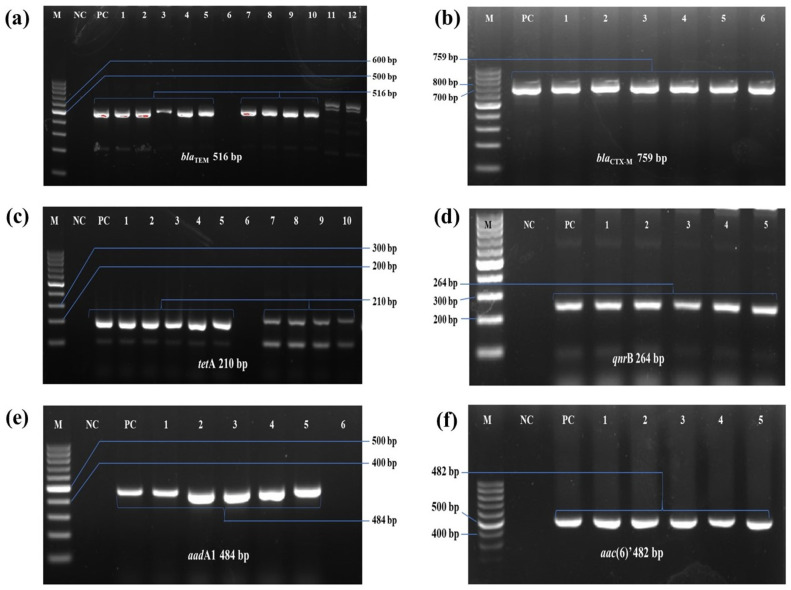
PCR amplification result of antibiotic resistance gene detection of *E. tarda.* PCR amplification of antibiotic resistance genes in *E. tarda* isolates. (a) *bla*_TEM_ 516 bp; (b) *bla*_CTX-M_ 759 bp; (c) *tet*A 210 bp; (d) *qnr*B 264 bp; (e) *aad*A1 484 bp; (f) *aac*(6)’ 482 bp. In all cases, M: 100 bp molecular marker; NC: negative control; PC: positive control; absence of band or not in desired position, was considered as negative; positive isolates were indicated in the figures.

Molecular detection revealed most of isolates carried at least one of antibiotic resistance genes (ARGs), while only four (GF9-S, MF5-I, MF16-I, and GF1-L) being negative. Several strains harbored multiple ARGs simultaneously (**Table 4**). Among the detected genes, *bla*_TEM_ and *tet*A were the most prevalent, presented in 14 (43.75%) and 20 (62.5%) of isolates, Remaining genes were detected at lower frequencies: *bla*_CTX-M_ (28.1%, 9/32), *qnr*B (21.9%, 7/32), *aad*A1 (46.9%, 15/32), and *aac*(6)’ (28.13%, 9/32) (Fig 4).

**Table 4:** Phenotypic resistance, multiple antibiotic resistance (MAR) index, resistance genes, and virulence associated gene profile of *E. tarda* isolates.

*E. tarda* isolates	Sample types	Phenotypic resistance patterns	MAR index	Virulence genes				Antibiotic resistance genes (ARGs)					
				*muk*F	*gad*B	*fim*A	*cit*C	*bla*_TEM_	*bla*_CTX-M_	*tet*A	*tet*B	*aad*A1	*aac*(6)’
TF2-S	Skin	AMX, COT	0.15	●	●	●	+	+	●	+	●	+	●
TF5-S	Skin	AMX, AZM, LEV, NA, CTR, O, S, K	0.61	+	+	●	+	+	+	+	●	+	+
TF7-S	Skin	AMX, AZM, NA, O, COT	0.38	●	●	●	●	●	●	●	+	●	●
TF11-S	Skin	AMX, NV	0.15	●	+	+	●	+	●	+	●	●	+
MF3-S	Skin	AMX, LEV, NA, O, S, K, COT	0.53	●	●	●	●	+	●	+	●	+	●
MF4-S	Skin	AMX, AZM, NA, O, COT	0.38	+	●	●	●	+	+	+		+	●
MF6-S	Skin	AMX, NV, K, COT	0.31	●	●	●	●	+	●	+	+	+	●
MF8-S	Skin	AMX, AZM, NA, O, COT	0.38	+	●	+	●	+	●	+	●	●	●
MF16-S	Skin	AMX, AZM, CTR, O, K, COT, ATM	0.53	+	●	●	●	+	●	+	+	+	●
GF2-S	Skin	AMX, O, COT, ATM	0.31	+	●	●	+	●	+	●	●	●	●
GF9-S	Skin	AMX, AZM, NA, K, COT, ATM	0.46	●	●	●	●	●	●	●	●	●	●
GF10-S	Skin	AMX, NV, NA, O, COT	0.38	●	●	●	●	●	●	●	+	●	●
TF1-I	Intestine	AMX, AZM, NA, O, COT	0.38	●	+	●	●	●	●	●	●	●	+
TF4-I	Intestine	AMX, AZM, CTR, O, K, COT, ATM	0.53	●	●	●	●	+	●	+	●	●	●
TF11-I	Intestine	AMX, LEV, CTR, O, S, K, COT, ATM	0.61	+	+	●	●	+	+	+	●	+	+
MF3-I	Intestine	AMX, LEV, S, K, COT, ATM	0.46	+	●	●	●	●	+	+	●	+	●
MF6-I	Intestine	AMX, AZM, CTR, O, K, COT, ATM	0.53	●	●	●	●	●	●	+	●	●	●
MF5-I	Intestine	AMX, AZM, O, COT	0.31	+	●	●	●	●	●	●	●	●	●
MF10-I	Intestine	AMX, AZM, K, COT, ATM	0.38	●	●	●	●	+	●	+	●	+	●
MF12-I	Intestine	AMX, AZM, LEV, O, K, COT	0.46	+	+	●	●	●	+	●	+	●	+
MF16-I	Intestine	AMX, AZM, LEV, NV, NA, CTR, COT, ATM	0.61	+	●	●	●	●	●	●	●	●	●
GF5-I	Intestine	AMX, AZ, LEV, NA, O, K, COT	0.53	●	●	●	●	●	●	●	+	●	●
GF9-I	Intestine	AMX, AZM, NV, COT	0.31	●	+	●	●	+	+	+	●	+	●
GF10-I	Intestine	AMX, AZM, CTR, O, S, COT, ATM	0.53	●	+	●	●	+	●	+	●	+	+
TF1-L	Liver	AMX, AZM, K, COT, ATM	0.38	●	●	●	●	●	●	+	●	●	●
TF5-L	Liver	AMX, LEV, COT, ATM	0.31	●	●	+	●	●	●	+	●	+	+
TF11-L	Liver	K, COT, ATM	0.23	+	●	+	+	●	+	●	●	●	+
MF4-L	Liver	AMX, AZM, LEV, NV, NA, O, S, COT	0.61	+	●	●	●	●	●	+	●	+	●
MF6-L	Liver	AZM, O, K, COT	0.31	●	●	●	●	●	+	+	●	+	●
MF8-L	Liver	AMX, NV, O, K, COT, ATM	0.46	+	●	●	●	●	●	●	●	●	+
MF12-L	Liver	AMX, AZM, LEV, O, S	0.38	●	●	●	●	+	●	+	+	+	●
GF1-L	Liver	AMX, AZM, LEV, NA, CTR, K, COT	0.46	+	●	●	+	●	●	●	●	●	●

**Legends**: AMX: Amoxicillin; S: Streptomycin; K: Kanamycin; CRO: Ceftriaxone; NV: Novobiocin; LEV: Levofloxacin; ATM: Aztreonam; CN: Gentamicin; NA: Nalidixic acid; MEM: Meropenem; AZM: Azithromycin; COT: Cotrimoxazole; OT: Oxytetracycline; +: presence; ●: absence; TF: fish sample from Trishal upazila; MF: fish sample from Muktagachha upazila; GF: fish sample from Gouripur upazila

### 3.6. MDR patterns and MAR index analysis of the isolated *E. tarda*

The MDR patterns analysis revealed that 30 (93.75%) isolates were found as MDR. Among 30 MDR isolates, 7 (21.88%) isolates showed resistance against 7 classes of antibiotics, 6 isolates against 6 classes, 10 isolates against 5 classes, 6 isolates against 4 classes, and one toward three classes (Table 4). The MAR index ranged from 0.15 to 0.61, while most of the isolates exceed the threshold value 0.2. The average MAR index for all the tested isolates was 0.381 (Table 4).

### 3.7. Distribution of virulence genes

**Fig 5:**
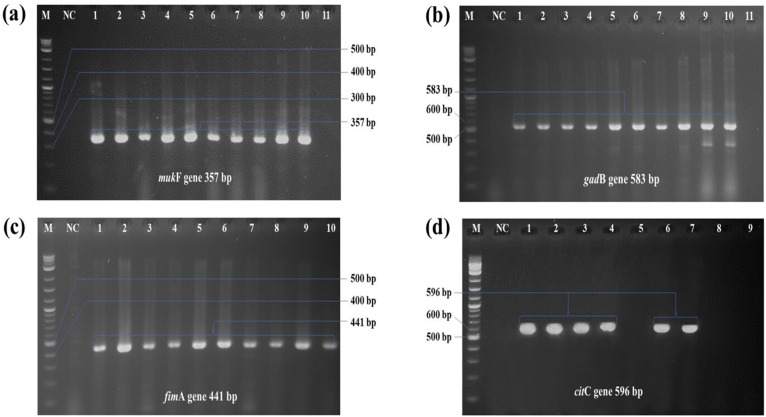
PCR amplification result of virulence gene detection of *E. tarda.* PCR amplification for virulence associated genes of *E. tarda;* a) lane 1–9 positive amplicon at 357 bp for *muk*F gene, b) lane 1–7 positive amplicon at 583 bp for *gad*B gene, c) lane 1–4 positive amplicon at 441 bp for *fim*A gene, and d) lane 1–4 positive amplicon at 596 bp for *cit*C gene (M = 100 bp DNA ladder; Lane NC: negative control).

Out of 32 strains of *E. tarda*, a total of 14 isolates were found positive *mukF* gene. The overall occurrence of *muk*F gene was 43.8%. In case of *gad*B gene, a total 17.5% (7/32), *fim*A gene 12.5% (4/32), *cit*C gene 15% (5/32) stain found positive (Fig 5), respectively. Co-existence found in two strains contain (*muk*F, *gad*B, *cit*C) and (*muk*F, *fim*A, *cit*C) genes. Two genes present in four isolates, others contain only one gene (**Table 4**). Among 32 isolates, 12 were found negative for any target virulence associated genes (**Table 4**).

### 3.8. Relation between phenotypic resistance, genotypic resistance, and virulence factors of isolated *E. tarda*

Bivariate analysis revealed a negative significant correlation between the *bla*_CTX-M_ and amoxicillin (r = −0.413, p = 0.019) and positive significant correlation between *bla*_CTX-M_ and kanamycin (r = 0.588, p = 0.000) was observed (**Table 5**). Again, bivariate analysis between virulence genes and antibiotic resistance genes showed strong positive association between *gad*B and *aac*(6)’ (r = 0.678, *p* = 0.000), *bla*_TEM_ and *tet*A (r = 0.683, p = 0.000), *bla*_TEM_ and aadA1 (r = 0.560, p = .002) (**Table 6**). However, moderate significant correlation were also observed between *muk*F and *bla*_CTX-M_ (r = 0.429, p = 0.017) and *fim*A and *aac*(6)’ (r = 0.394, p = 0.028) (**Table 6**).

**Table 5:** Correlation between the phenotypic and genotypic antibiotic resistance patterns.

ARGs	Phenotypic resistance profile												
	AMX	AZM	LEV	NV	NA	CTR	O	S	K	CN	COT	MEM	ATM
*bla*_TEM_	−0.033	−0.025	−0.240	−0.262	−0.293	0.218	0.033	−0.101	0.071	–	−0.149	–	0.238
*bla*_CTX-M_	−0.413*	0.160	0.133	0.301	−0.054	0.120	0.054	0.056	0.588**	–	−0.037	–	−.149
*tet*A	0.067	−0.153	0.119	0.289	−0.333	0.149	0.200	0.041	0.049	–	−0.249	–	0.293
*tet*B	0.137	0.065	0.254	−0.061	0.059	−0.131	0.098	0.133	−0.260	–	0.170	–	−0.010
*aad*A1	−0.016	−0.243	−0.021	0.291	−0.210	0.036	0.081	0.030	0.129	–	0.087	–	0.308
*aac*(6)’	0.162	−0.133	−0.160	0.234	−0.197	−0.040	−0.090	−0.122	0.170	–	−0.276	–	0.009

**Legends:** AMX: Amoxicillin; S: Streptomycin; K: Kanamycin; CRO: Ceftriaxone; NV: Novobiocin; LEV: Levofloxacin; ATM: Aztreonam; CN: Gentamicin; NA: Nalidixic acid; MEM: Meropenem; AZM: Azithromycin; COT: Cotrimoxazole; OT: Oxytetracycline

**Table 6:** Spearman’s correlation between virulence associated genes and resistance genes in *E. tarda* isolates.

	Correlations									
	*muk*F	*gad*B	*fim*A	*cit*C	*bla*_TEM_	*bla*_CTX-M_	*tet*A	*tet*B	*aad*A1	*aac*(6)’
*muk*F	NA	−.010	.048	.314	−.143	.429^*^	−.228	−.162	.071	.149
*gad*B		NA	.029	−.020	.295	.342	.098	−.097	.109	.678^**^
*fim*A			NA	.098	.048	−.026	.098	−.200	−.166	.394^*^
*cit*C				NA	−.033	.305	−.200	−.228	−.059	.114
*bla*_TEM_					NA	.009	.683^**^	−.010	.560^**^	.009
*bla*_CTX-M_						NA	.054	−.163	.248	.227
*tet*A							NA	−.215	.728^**^	−.090
*tet*B								NA	−.043	−.163
*aad*A1									NA	.030
*aac*(6)’										NA

**Note**: Statistical analysis between virulence factor and resistance gene. P-value was calculated by spearman’s ranks correlation test.

* Correlation is significant at 0.05 level (2-tailed)

**Correlation is extremely significant at 0.01 level (2-tailed).

Abbreviation: NA, not applicable.

## 4. Discussion

*Edwardsiella tarda* is an important pathogen in aquaculture, causes edwardsiellosis in catfish and contribution to substantial mortality and economic loses worldwide [45,46]. The clinical signs observed in infected catfish resembling *Aeromonas hydrophila* infections; however, haemolysis serves a distinguishing feature [47]. This bacterium is widely distributed among freshwater and marine fish, as well as of diverse aquatic environment, reflecting its’ ecological adaptability [48,49]. While primarily known as animal pathogen [50], *E. tarda* poses zoonotic threat, cause gastrointestinal [51] and extra-intestinal infection including wound infections, bacteraemia, septic arthritis, and muscle necrosis [52–55].

The overall prevalence of *E. tarda* in stinging catfish (*Heteropneustes fossilis*) is this study (47.5%) was higher than previously reported in Bangladesh [7], Uganda (8.3%) from *Clarias gariepinus* (African catfish) [56], and Ethiopia (7.6%) from tilapia samples [57]. Area-specific prevalence range from 45% to 53% indicating that local aquaculture practice and environmental condition may influence pathogen distribution as Manzoor et al. [7] observed same differences.

Observed pathological sign including petechial haemorrhages on the skin and fins, along with abdominal fluid accumulation (ascites) are consistent with clinical manifestation of Edwardsiellosis in catfish [58,59], highlighting the clinical relevance of this pathogen. However, the detection of both symptomatic and asymptomatic carriers underscores the potential for horizontal transmission among the aquatic species and the importance of confirmatory detection for effective disease management [60].

Phenotypic characterization of the isolates including colony morphology, growth on selective agar media and biochemical profile were largely consistent with previously reported *E. tarda* isolated from cultured freshwater tilapia, African catfish, Chinook salmon, and sharp snout sea bream [61,62]. The Gram’s staining results showed the presence of Gram-negative bacteria with a coccobacillary or short rod-shaped morphology [63]. Regarding the biochemical findings, the isolates showed positive results for the Methyl Red (MR) and Catalase tests, while results for the Voges-Proskauer (VP) and Indole tests were negative. Additionally, the isolates were capable of fermenting basic sugars, *viz*., glucose, maltose, mannitol, dextrose, and sucrose, and produced acid. These biochemical profiles are generally consistent with findings reported in previous studies on *E. tarda* [61,63]. However, a discrepancy was noted in the Indole test results, only two isolates were Indole-positive, while the majority were negative. This deviation may suggest the presence of closely related *Edwardsiella* species, emphasizing the limitations of phenotypic methods for precise identification [64].

Molecular detection of microbial isolates provides superior specificity and reliability compared to conventional bacteriological methods, enabling precise detection and differentiation of target organisms, such as *E. tarda*, from closely related or concurrently infecting one at both the genus and species levels [62,64]. Ultimately, phenotypic criteria-based detection of *E. tarda*, in some instances, may show false-positive results, involving closely-related genera. To address this limitation, polymerase chain reaction (PCR) was explored, targeting the genes specific to the *Edwardsiella* genus, *viz*., *gro*EL, *etf*A, *etf*D, and *gyr*B. Among these primer sets, only *gro*El gene -based primer set was new and designed. *gro*EL is one of the house-keeping genes and proved itself as suitable molecular marker for PCR detection of bacterial pathogens [30].

In this study, four recognized *E. tarda* virulence-associated genes including *cit*C, *muk*F, *gad*B, and *fim*A, were detected. These genes are associated with critical pathogenic mechanisms, including adhesion; intracellular persistence, cytotoxicity, and evasion of host immune responses [46,65]. Particularly, *fim*A facilitate the binding of the bacterial cell with the specific host-cell receptors and influencing tissue tropism; while *gad*B and *kat*B are contribute to bacterial survival against host’s phagocytic mechanism [66,67]. Among these, *muk*F and *gad*B had also been identified by Nagy et al. (2018) and are considered as virulence determinants of pathogenic *E. tarda* strains. In the present study, prevalence of these genes among the isolates was 43.8% *muk*F gene, 17.5% *gad*B, 12.5% *fim*A gene, and 15% *cit*C.

Virulence factors, identified in this study suggested that *E. tarda* isolates are capable of establishing systemic infection under favourable environmental condition. Surface structures that facilitate motility, adhesion, and host-pathogen interaction further reinforce pathogenic potential of these strains [68]. Although this study did not evaluate the isolate’s virulence through *in vivo* and *in vivo* testing, the presence of these virulence-associated genes, along with the observed clinical symptoms, suggests the potential occurrence of edwardsiellosis in local aquaculture systems.

Antibiotic sensitivity of the isolated strains revealed complete sensitivity towards gentamicin and meropenem, found 100% effective for treatment of edwardsiellosis. These results are consistent with Manzoor et al. [7] who also reported full sensitivity to gentamicin. High resistance also observed against Cotrimoxazole and Amoxicillin (S3 **Table**). Oxytetracycline, Azithromycin and Kanamycin were found 62.5%, 65.6% and 59.6% resistance for the isolates. Report of Abd El Tawab et al. [69] showed 50% and 75% sensitivity towards Streptomycin and Ceftriaxone, respectively. Again, 100% resistance *E. tarda* to Amoxicillin was also reported by Ogbonne et al. [70] and Abd El Tawab et al. [69]. This study found 62% resistance towards Oxytetracycline which is far different from that reported by Abd El Tawab et al. [69] but close finding was reported by Manzoor et al. [7] as 50%. The resistance profile of the isolates against Nalidixic acid and Trimethoprim-sulfamethoxazole were consistent with findings of Akinbowale et al. [71]. In contrast, the findings of this study differ from those of Ogbonne et al. [70], who reported *E. tarda* isolates to be resistant to amoxicillin and nalidixic acid, while exhibiting intermediate susceptibility to gentamicin. Concerning antibiotic resistance genes (ARGs), Abd El Tawab et al. [69] identified the presence of β-lactamase genes (*bla*_TEM_, *bla*_CTX-M_), aminoglycoside resistance gene (*aad*A1), and tetracycline resistance gene (*tet*A) in all *E. tarda* isolates obtained from *Clarias gariepinus* sampled from fish farms and local markets in the Kafrelsheikh governorate of Egypt. Manzoor et al. [7] also found *bla*_TEM_ gene in *E. tarda* isolates from *Oreochromis* spp. in Pakistan.

In the present study, 93.75% isolates were found as multi-drug resistant (MDR) which was in close agreement with the findings of Samir et al. [72] who found 100% isolates of *E. tarda* as MDR from Nile tilapia collected from Egyptian fish farms. Besides, based on the data presented in the Table 4, it can be assumed that the fish were highly exposed to antibiotics frequently used in aquaculture, as all the isolates exhibited an multiple-antibiotic resistance (MAR) index ranging from 0.15 to 0.61 with average value of.392 MAR index, as per description of Nyandjou et al. [43]. A similar finding was reported by Abd El Tawab et al. [69] where the highest MAR index value was.538 (2 isolates) and lowest was 0.154 (eight isolates) with average value of 0.327 for all tested isolates.

The widespread resistance of many Gram-negative bacteria to β-lactam antibiotics is largely due to their inherent resistance mechanisms, which are often encoded on the chromosome and can be inherited by future generations [73]. Additionally, the heavy use of tetracyclines, particularly as growth enhancers in animal farming and aquaculture, has led to the discovery of oxytetracycline resistance genes (*tet*). These genes are associated with several mechanisms, including membrane-bound active efflux systems, ribosome-associated resistance factor, and enzymes capable of inactivating tetracyclines [74]. The present study observed the presence of the β-lactam resistance determinants, *viz*., *bla*_TEM_ *bla*_CTX-M_ were observed in 43.75% 28.13% of the total isolates, respectively, which aligns with findings from Manzoor et al. [7] and Wimalasena et al. [18]. The high prevalence may be due to their chromosomal-mediated, intrinsic resistance and rapid transmission to the next generations of pathogenic bacteria [73]. Notably, the aminoglycoside resistance genes *aac*(6´)-Ib and *aad*A1 were most commonly found in the current *E. tarda* isolates, with the majority exhibiting multidrug resistance, similar to the findings reported by Wimalasena et al. [18].

The therapeutic, prophylactic and metaphylactic use antimicrobial agents in aquaculture system contribute to the development of antimicrobial resistance among aquatic pathogens. Interestingly, the findings of this study indicate a low level of acquired antibiotic resistance, consistent with the observations reported by Wamala et al. [56]. Analysing antibiogram profiles is valuable for predicting future trends in antibiotic resistance. However, these profiles often vary across different studies, likely due to strain-specific characteristics and varying environmental selective pressures [75]. As there is no alternative to antibiotics to control the infectious disease outbreaks in aquaculture system, particularly in the context of developing countries, like Bangladesh, the findings of the present study reflected the alarming scenario of resistance levels of *Edwardsiella* to antimicrobials, as well as their virulence profile. In the context of Bangladesh, there are still huge scopes of researches in this field.

Nevertheless, certain limitations of this study should be acknowledged. The relatively small sample size restricts the generalizability of prevalence and resistance trends across border region, emphasizing the need for large-scale, multi-farm investigations. Moreover, while molecular detection of virulence provides valuable insight, the absence of in vivo challenges experiments limits the ability to confirm actual pathogenic potential of the isolates. Future research should therefore incorporate controlled infection models to better ink between gene presence and disease severity.

## 5. Conclusion

This study reveals the significant presence of multidrug-resistant *E. tarda* in stinging catfish, posing a potential public health threat. Given the high resistance to common antibiotics, the findings highlight the urgent need for improved surveillance and strict biosecurity and management settings to prevent the spread of these resistant strains, particularly in regions with high incidence rates.

## Supporting information

S1 Fig Growth of suspected *Edwardsiella* spp. on different agar media. a. NA (colorless, watery, smooth colonies); b. SS agar (Small colonies with black centers); c. MacConkey agar media (pale color colony); d. EMB agar media (Pale pink colored, moist, glistening colonies); e. ET-Agar media with Colistin-sulphate; f. ET-Agar media without Colistin sulphate (Clear to whitish with black center colonies); g. NA; SS-agar media; ET-agar media with Colistin sulphate and without Colistin sulphate h. Bovine blood agar (beta type haemolysis).
(TIF)

S1 Table Total number of stinging catfish samples collected from sampling areas. (DOCX)

S2 Table Phenotypic characterization result of *E. tarda* using various bacteriological media. (DOCX)

S3 Table Results of antibiogram of *E. tarda* isolates from stinging catfish samples. (DOCX)
