# Supplementary figures and images for: Virulent MDR Edwardsiella tarda from stinging catfish (Heteropneustes fossilis)
Source: PLoS One. 2026 Jan 30;21(1):e0340061. doi: 10.1371/journal.pone.0340061 (PMC12857957; doi:10.1371/journal.pone.0340061)

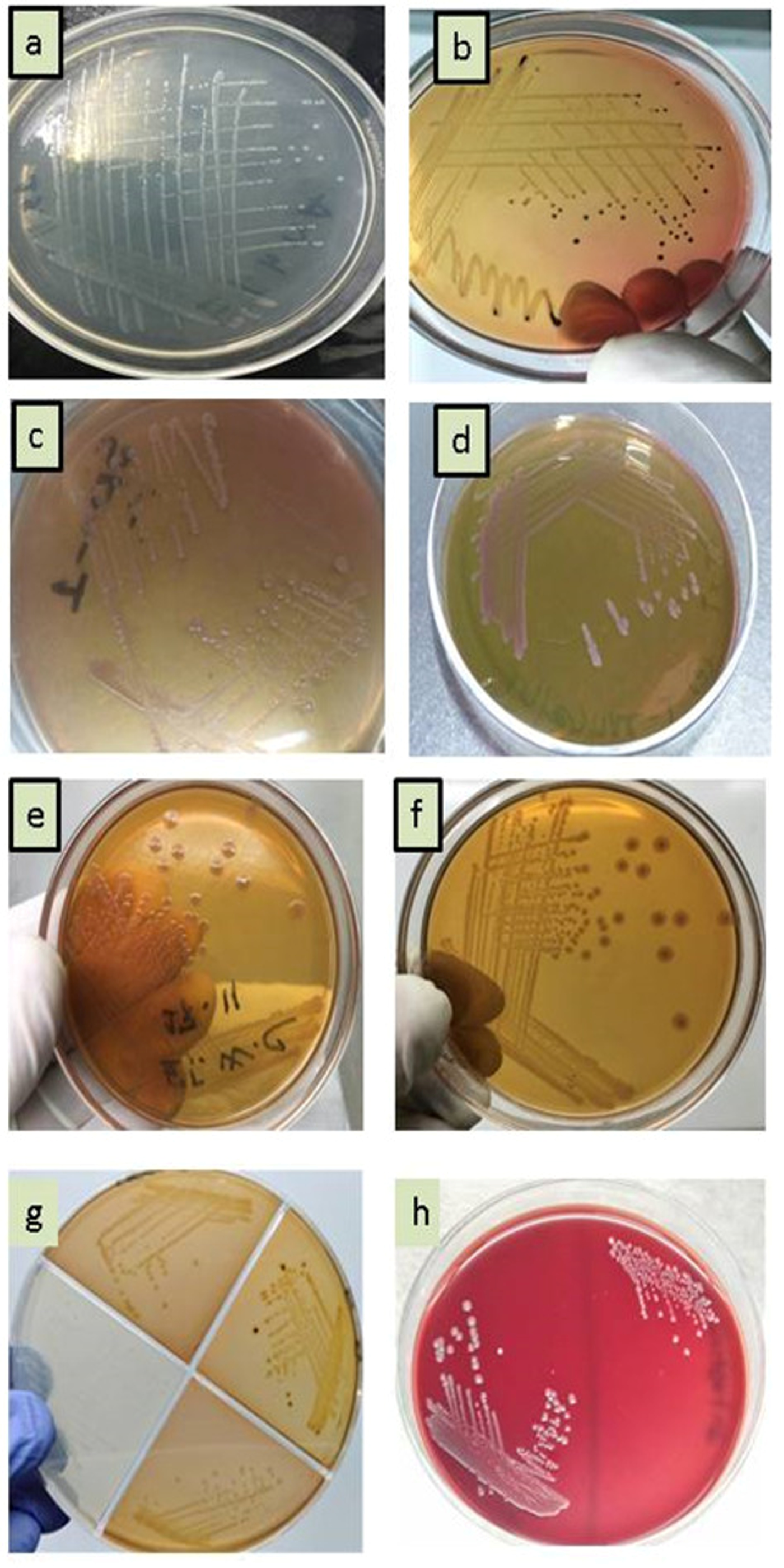

Supplement: S1 Fig — NA (colorless, watery, smooth colonies); b. SS agar (Small colonies with black centers); c. MacConkey agar media (pale color colony); d. EMB agar media (Pale pink colored, moist, glistening colonies); e. ET-Agar media with Colistin-sulphate; f. ET-Agar media without Colistin sulphate (Clear to whitish with black center colonies); g. NA; SS-agar media; ET-agar media with Colistin sulphate and without Colistin sulphate h. Bovine blood agar (beta type haemolysis). (TIF) [file pone.0340061.s001.tif]
