# Supplementary material for: Virulent MDR Edwardsiella tarda from stinging catfish (Heteropneustes fossilis)
Source: PLoS One. 2026 Jan 30;21(1):e0340061. doi: 10.1371/journal.pone.0340061 (PMC12857957; doi:10.1371/journal.pone.0340061)
Supplement: S1 Table — (DOCX) [file pone.0340061.s002.docx]

**S1 Table**: Total number of stinging catfish samples collected from sampling areas

| **Sampling location** | **Total samples** | **Nature of the samples** | **No. of desired samples** | **Total** |
| --- | --- | --- | --- | --- |
| **Trishal** | 12 | Skin | 12x4 | 48 |
|  |  | Intestine |  |  |
|  |  | Liver |  |  |
|  |  | Kidney |  |  |
| **Muktagachha** | 17 | Skin | 17x4 | 68 |
|  |  | Intestine |  |  |
|  |  | Liver |  |  |
|  |  | Kidney |  |  |
| **Gouripur** | 11 | Skin | 11x4 | 44 |
|  |  | Intestine |  |  |
|  |  | Liver |  |  |
|  |  | Kidney |  |  |
| **Total** | **40** |  |  | **160** |
