# Supplementary material for: Virulent MDR Edwardsiella tarda from stinging catfish (Heteropneustes fossilis)
Source: PLoS One. 2026 Jan 30;21(1):e0340061. doi: 10.1371/journal.pone.0340061 (PMC12857957; doi:10.1371/journal.pone.0340061)
Supplement: S2 Table — (DOCX) [file pone.0340061.s003.docx]

**S2 Table:** Phenotypic characterization result of *E. tarda* using various bacteriological media

| **Cultural Characterization** | | **Biochemical Characterization** | | | | | | |
| --- | --- | --- | --- | --- | --- | --- | --- | --- |
| **Culture Media** | **Colony characteristics** | **Catalase** | **Motility** | **Sugar** | | **MR** | **VP** | **Indole** |
| Nutrient agar | Colorless, watery, smooth colonies | + | + | Dextrose | + | + | - | + |
| MacConkey agar | Pale colored colonies |  |  | Maltose | + |  |  |  |
| Salmonella-Shigella agar | Small colonies with black centers |  |  | Lactose | - |  |  |  |
| Eosin methylene blue (EMB) | Pale pink, moist, glistening colonies |  |  | Sucrose | + |  |  |  |
| ET-agar with Colistin-sulphate and without Colistin-sulphate | Clear to whitish with black Centre colonies |  |  | Mannitol | - |  |  |  |
| Bovine Blood Agar | Beta type hemolysis |  |  |  |  |  |  |  |
