# Supplementary material for: Virulent MDR Edwardsiella tarda from stinging catfish (Heteropneustes fossilis)
Source: PLoS One. 2026 Jan 30;21(1):e0340061. doi: 10.1371/journal.pone.0340061 (PMC12857957; doi:10.1371/journal.pone.0340061)
Supplement: S3 Table — (DOCX) [file pone.0340061.s004.docx]

**Table S3:** Results of antibiogram of *E. tarda* isolates from stinging catfish samples

| **Sample ID** | **Antibiotics** | | | | | | | | | | | | |
| --- | --- | --- | --- | --- | --- | --- | --- | --- | --- | --- | --- | --- | --- |
|  | **AMX** | **AZM** | **LEV** | **NV** | **NA** | **CTR** | **O** | **CN** | **S** | **K** | **COT** | **MEM** | **ATM** |
| **TF2-S** | R | I | S | S | S | S | S | S | I | I | R | S | S |
| **TF5-S** | R | R | R | S | R | R | R | S | R | R | S | S | S |
| **TF7-S** | R | R | S | S | R | S | R | S | I | S | R | S | S |
| **TF11-S** | R | I | S | R | S | S | S | S | S | S | S | S | S |
| **TF1-I** | R | R | S | S | R | S | R | S | I | S | R | S | S |
| **TF4-I** | R | R | S | I | S | R | R | S | I | R | R | S | R |
| **TF11-I** | R | S | R | I | I | S | S | S | I | R | R | S | R |
| **TF1-L** | R | R | S | I | S | R | R | S | I | R | R | S | R |
| **TF5-L** | R | S | R | I | I | S | S | S | S | S | R | S | R |
| **TF11-L** | I | I | S | I | S | S | S | S | S | R | R | S | R |
| **MF3-S** | R | I | R | I | R | S | R | S | R | R | R | S | S |
| **MF4-S** | R | R | S | S | R | S | R | S | I | S | R | S | S |
| **MF6-S** | R | I | S | R | S | S | S | S | S | R | R | S | S |
| **MF8-S** | R | R | S | S | R | S | R | S | I | I | R | S | S |
| **MF16-S** | R | R | S | I | S | R | R | S | I | R | R | S | R |
| **MF3-I** | R | S | R | I | I | S | S | S | R | R | R | S | R |
| **MF6-I** | R | R | S | I | S | R | R | S | I | R | R | S | R |
| **MF5-I** | R | R | S | I | I | S | R | S | S | I | R | S | S |
| **MF10-I** | R | R | S | I | I | S | S | S | S | R | R | S | R |
| **MF12-I** | R | R | R | I | S | S | R | S | I | R | R | S | S |
| **MF16-I** | R | R | R | R | R | R | S | S | S | S | R | S | R |
| **MF4-L** | R | R | R | R | R | S | R | S | R | I | R | S | S |
| **MF6-L** | I | R | S | S | S | S | R | S | I | R | R | S | S |
| **MF8-L** | R | S | S | R | I | S | R | S | I | R | R | S | R |
| **MF12-L** | R | R | R | I | S | I | R | S | R | S | S | S | S |
| **GF2-S** | R | S | S | I | S | S | R | S | I | S | R | S | R |
| **GF9-S** | R | R | S | I | R | S | S | S | I | R | R | S | R |
| **GF10-S** | R | I | S | R | R | S | R | S | S | S | R | S | S |
| **GF5-I** | R | R | R | I | R | S | R | S | I | R | R | S | S |
| **GF9-I** | R | R | S | R | I | I | S | S | I | I | R | S | S |
| **GF10-I** | R | R | S | I | S | R | R | S | R | I | R | S | R |
| **GF1-L** | R | R | R | I | R | R | S | S | I | R | R | S | S |

**Legends**: TF: indicates fish samples from Trishal; MF: indicates fish samples from Muktagachha; GF: indicates fish samples from Gouripur; S: sensitive; R: resistant; I: Intermediately resistance; AMX: Amoxicillin; S: Streptomycin; K: Kanamycin; CTR: Ceftriaxone; NV: Novobiocin; LEV: Levofloxacin; ATM: Aztreonam; CN: Gentamicin; NA: Nalidixic acid; MEM: Meropenem; AZM: Azithromycin; COT: Cotrimoxazole; OT: Oxytetracycline; TF: fish samples from Trishal upazila; MF: fish samples from Muktagachha upazila; GF: fish samples from Gouripur upazila.
